# An experimental investigation on the effect of beam angle optimization on the reduction of beam numbers in IMRT of head and neck tumors

**DOI:** 10.1120/jacmp.v13i4.3912

**Published:** 2012-07-05

**Authors:** V. K. Sathiya Narayanan, R. Vaitheeswaran, Janhavi R. Bhangle, Sumit Basu, Vikram Maiya, Bhooshan Zade

**Affiliations:** ^1^ Department of Radiation Oncology Ruby Hall Clinic Pune India; ^2^ Healthcare Sector Siemens Ltd MG Road Nungambakkam Chennai India

**Keywords:** beam number, beam angles, IMRT, radiotherapy, inverse planning

## Abstract

In static intensity‐modulated radiation therapy (IMRT), the fundamental factors that determine the quality of a plan are the number of beams and their angles. The objective of this study is to investigate the effect of beam angle optimization (BAO) on the beam number in IMRT. We used six head and neck cases to carry out the study. Basically the methodology uses a parameter called “Beam Intensity Profile Perturbation Score” (BIPPS) to determine the suitable beam angles in IMRT. We used two set of plans in which one set contains plans with equispaced beam configuration starting from beam numbers 3 to 18, and another set contains plans with optimal beam angles chosen using the in‐house BAO algorithm. We used quadratic dose‐based single criteria objective function as a measure of the quality of a plan. The objective function scores obtained for equispaced beam plans and optimal beam angle plans for six head and neck cases were plotted against the beam numbers in a single graphical plot for effective comparison. It is observed that the optimization of beam angles reduces the beam numbers required to produce clinically acceptable dose distribution in IMRT of head and neck tumors. Especially $N_{0.1}$ (represents the beam number at which the objective function reaches a value of 0.1) is considerably reduced by beam angle optimization in almost all the cases included in the study. We believe that the experimental findings of this study will be helpful in understanding the interplay between beam angle optimization and beam number selection process in IMRT which, in turn, can be used to improve the performance of BAO algorithms and beam number selection process in IMRT.

PACS number: 87.55.de

## I. INTRODUCTION

In the current practice of radiotherapy, intensity‐modulated radiation therapy (IMRT) has become an inevitable tool for radical cure of cancer with minimal side effects to the surrounding normal tissues. Currently, there are many forms of IMRT, which can be broadly classified into: 1) static IMRT (sIMRT), and 2) rotational IMRT (rIMRT). In static IMRT (either segment‐based or dynamic IMRT), the fundamental factors that determine the quality of a plan are the number of beams and their angles. It is well established that optimization of beam angles in IMRT is very useful in generating better treatment plans.^(^ ^1^ ^,^ ^2^ ^)^ Currently, there exist a number of beam angle optimization (BAO) algorithms, which give case‐specific solutions to the beam angle problem.^(^ ^3^ ^–^ ^7^ ^)^ Similarly there have been attempts to give approximate solutions to the beam number selection problem in IMRT, either by studying the effect of beam numbers in the quality of IMRT plan^(^ ^8^ ^,^ ^9^ ^)^ or approaching the problem from fundamental theoretical viewpoints.^(^ ^10^ ^,^ ^11^ ^)^

Prior to optimizing the beam angles, many of the current BAO algorithms require the input of number of beams for a given plan. On the other hand, investigations showed that optimization of beam angles can possibly reduce the total number of beams used in IMRT. The interplay between beam angle optimization and beam number selection for coplanar IMRT was initially investigated by Soderstrom and Brahme.^(^ ^12^ ^)^ This study showed that a plan with fewer number of beams with optimized angles can be better than or equal to that of plans with many equispaced beams. The objective of this study is to investigate the effect of beam angle optimization on the beam numbers in detail for head and neck tumors.

## II. MATERIALS AND METHODS

### A. Brief description of the study

We used six head and neck cases in the study. Essentially our methodology uses a parameter called “Beam Intensity Profile Perturbation Score (BIPPS)” to determine the suitable beam angles in IMRT, which was introduced in our previous paper.^(^ ^13^ ^)^ Two set of plans were used in which one set contains plans with equispaced beam configuration starting from beam numbers 3 to 18, and another set contains plans with optimal beam angles chosen using the in‐house beam angle optimization algorithm. The beam angle in equispaced beam geometry is given by:

(1) $$\theta_{i}=\left(\frac{360}{N}(i-1)+\theta_{1}\right)\mathrm{mod}\;360$$

where $\theta_{i}$ is the gantry angle of beam i, and *N* is the number of beams. We used dose‐based objective function as a measure of the quality of a plan. The objective function scores obtained for equispaced beam plans and optimal beam angle plans for the six cases were plotted against the beam numbers in a single graphical plot for effective comparison. The following sections describe the study methods in more detail.

### B. Beam intensity profile perturbation score (BIPPS)

In our earlier paper,^(^ ^13^ ^)^ we introduced the parameter BIPPS to select suitable beam angles in IMRT. Basically BIPPS is a measure of how much the intensity profiles of a set of beams are perturbed in a plan when the normal dose‐volume constraints of the plan are resolutely tightened. Let the plan with normal dose‐volume constraints be called Plan A and the plan with tightened dose‐volume constraints be called Plan B. Then, the BIPPS scoring at any given beam angle Θ can be mathematically described as follows:

(2) $$\mathrm{BIPPS}(\theta )=\left(\frac{1}{\mathrm{nxm}}\right)\sum_{i=1}^{m}\sum_{j=1}^{m}{{[}^{\theta }P_{A}(i,j){-}^{\theta }P_{B}(i,j)]}^{2}$$

In Eq. (2), an intensity profile is considered as a 2D array of size n× m. Here, ${\;}^{\Theta }P_{A}$ is a relative intensity value of profile array A (in Plan A) at $i^{\text{th}}$ row and $j^{\text{th}}$ column at angle Θ, and ${\;}^{\Theta }P_{B}$ is the relative intensity value of profile array B (in Plan B) at $i^{\text{th}}$ row and $j^{\text{th}}$ column at the same angle.

It was demonstrated that in a given feasible beam set (0°–360°), those that have higher BIPPS values will be the suitable beam angles. Hence, a ranking function over the BIPPS parameter for all feasible beams will give the required number of suitable beam angles for the given case. One can interpret BIPPS as a measure of the relative freedom available for a beam to deliver a specific amount of dose to the target without exceeding the tolerance limits of surrounding normal structures. Hence, BIPPS will be specific to the patient anatomy and dose prescription.

### C. Case study description

The six head and neck cases chosen in the study have a wide range of complexity in terms of tumor size and shape, normal tissue locations, and dose constraints. In general, the proximity of spinal cord, and left and right parotids were the main constraints in all the cases. Additionally, left and right eyes were also included as critical organs. Table 1 gives the dose‐volume constraints for the six cases. In all the cases, the dose prescription to target volume was such that 95% of target volume should receive the clinician prescribed target dose.

**Table 1:** The dose‐volume constraints for the head and neck cases included in the study.

*Organ*	*Constraint Type*	*Dose (Gy)*	*Volume (%)*	*Priority*
PTV	Lower	50	100	300
	Upper	58	0	250
Spinal Cord	Upper	45	0	80
Parotids	Upper	23	0	60
Oral Cavity	Upper	17	0	20
Eyes	Upper	10	0	50
Normal Tissue	Upper	30	0	20
Skin	Upper	30	0	20

### D. Objective function

We used KonRad inverse planning system (Siemens AG, Munich, Germany) for the investigation. The objective of optimization is to minimize the difference between the prescribed and calculated dose distributions. In KonRad, the optimization is based on the following quadratic dose‐based objective function given by:

(3) $$F(d(x))=\sum_{i=1}^{n}[P_{i}{\left(D_{i}(x){‐}^{P}D_{i}\right]}^{2}$$

Here *F(d(x))* is the objective function, where *d(x)* is a dose distribution among the voxels of the patient model and *x* is the vector of parameters to be selected (beam weights) and *N* is the number of voxels. $D_{i}=D_{i}(x)$ and ${\;}^{p}Di$ are the calculated and prescribed maximum dose in voxel *i*, respectively. Here the array $P_{i}$ contains the penalties for different structures for violation of dose limits. For instance, a voxel in the target is penalized if it is overdosed ($>D_{\max}$), as well as underdosed ($<D_{\min}$). Likewise, a voxel in the normal structure get penalized if it receives a dose more than its $D_{\max}$.

It is clear from Eq. (2) that the lower the objective function value, the better would be the dose distribution to the target volume and normal structures. In single criteria optimization schemes, objective function can serve as an ideal indicator of the quality of IMRT plans.^(^ ^14^ ^)^ The objective function values were calculated for 3–18 equispaced beams and optimal beam angle configuration in each case. In order to make a meaningful comparison, the objective function parameters (OFPs) such as dose limits and tissue/organ penalties for target and OARs were kept the same in all plans involving different number of beams for a given case. The following are some of the important parameter settings used throughout the study: CT slice thickness=0.3 cm; Dose calculation grid size (pencil beam size) =0.3 cm in all directions; intensity levels used in the beamlet optimization =7 (for all six cases).

## III. RESULTS

**Figure 1:**
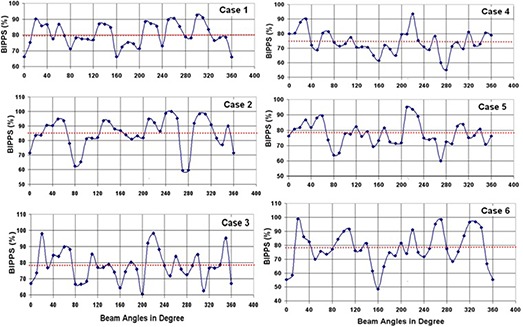
The percentage of Beam Intensity Profile Perturbation Score (%BIPPS) as a function of beam angles obtained for the head and neck cases included in the study.

**Figure 2:**
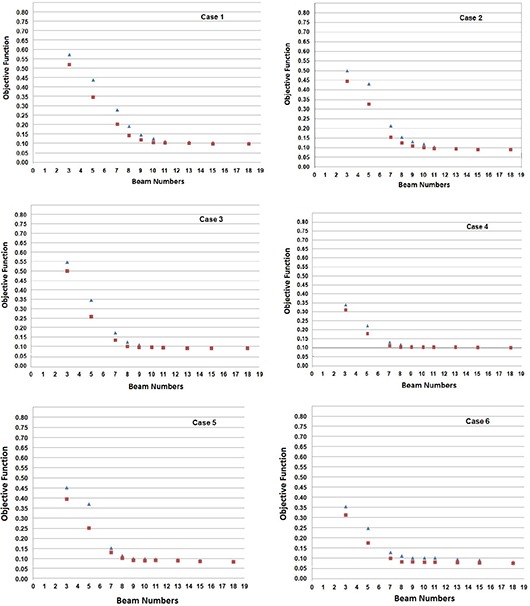
The plot of calculated objective function values at different beam numbers in optimal beam angle (square points) and equispaced beam angle (triangle points) configurations for the six cases ((a)–(f), respectively) in this study.

**Figure 3:**
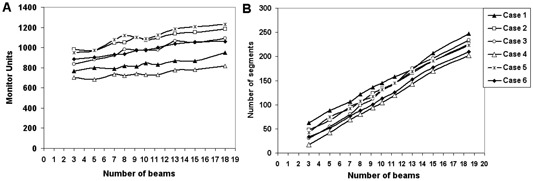
Plot of number of MUs (A) obtained at different beam numbers, and plot of total number of segments (B) obtained at different beam numbers.

**Figure 4:**
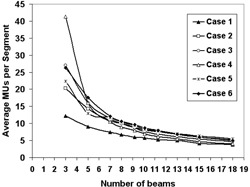
A plot of average MU per segment obtained at different number of beams.

In this study, we use a special parameter called $N_{0.1}$ for effective comparison of optimal beam angle and equispaced beam plans. $N_{0.1}$ represents the beam number at which the objective function reaches a value of 0.1. In our inverse planning system, we have observed that the final objective function value as low as 0.1 is desirable in order to get a clinically acceptable dose distribution. Figure 1 shows the BIPPS curves obtained for head and neck cases included in the study. As can be seen from the figure, there are many peaks in a given BIPPS curve, which in total includes 36 beams. As demonstrated in our previous study,^(^ ^13^ ^)^ the optimality of a given beam angle is directly proportional to its BIPPS value. Figure 2 is the plot of calculated objective function values (using Eq. (3)) at different beam numbers in optimal beam angle and equispaced beam angle configurations for each of the six cases (Figs. (a)–(f), respectively). Table 2 gives the values of $N_{0.1}$ obtained for both beam angle configurations. Figure 3 shows (A) the rise of monitor units (MUs) and (B) the number of segments with respect to the beam numbers. Figure 4 is the plot of average MU per segment obtained at different number of beams, which clearly shows that the average MU per segment considerably reduces with increasing number of beams. We have also observed that the curves in Figs. 3 and 4 are approximately the same for optimal beam angle and equispaced beam angle configurations.

**Table 2:** The comparison of $N_{0.1}$ obtained for optimal beam angle (Optimal) and equispaced beam angle (Equal) plans.

	$N_{0.1}$	
*Case No*	*Optimal*	*Equal*
1	10	11
2	10	11
3	8	10
4	8	10
5	8	9
6	7	9

## IV. DISCUSSION

Producing a good dose distribution with fewer number of total MUs and segments fundamentally requires an appropriate selection of number of beams and their angles. It has been reported that adding more beams in static IMRT beyond a point increases the MUs and number of segments without any considerable improvement in dose distribution, leading to more leakage radiation and increased critical organ dose.^(^ ^8^ ^)^ This is also evident from Figs. 3 and 4. The increase in MUs and segments may also lead to uncertainty in the treatment delivery. Moreover, as shown in Fig. 4, the average MU per segment rapidly decreases with increasing number of beams. This indicates that the probability of getting low MU segments can be very high with increased number of beams. Depending upon the dose rate and time taken for beam formation in a given linear accelerator, low MU segments can introduce significant errors in the dose delivery. These observations give a strong indication that a case‐specific selection of beam numbers has the potential to produce an optimal dose distribution with reduced errors during actual delivery. In the case of dynamic IMRT, more beams generally reduce MU per field, which will cause leaves to travel faster and, hence, introduce treatment delivery errors. It is evident from Fig. 2 and Table 2 that optimization of beam angles reduces the beam numbers required to produce clinically acceptable dose distribution. In particular, $N_{0.1}$ is considerably reduced by beam angle optimization in almost all the cases included in the study. The results indicate that BAO plays a crucial role in reducing total number of beams in IMRT.

The shape of the objective function curve is steep in the beginning and then starts saturating rapidly (Fig. 2). This is in line with the earlier findings on the effect of beam numbers in dose distribution.^(^ ^5^ ^)^ It is evident from the shape of the objective function curve that beyond a point, addition of beams in static IMRT (especially in segment‐based IMRT) contributes negligibly to the improvement of the dose distribution. The investigation using objective function is straightforward and also efficient, as the objective function is derived directly from the clinically appropriate dose‐volume indices of target volume and organs‐at‐risk (OARs).

The beam intensity levels (number of segments per beam) used in the optimization phase may also have a major impact on the beam numbers, especially in segment‐based IMRT. A plan with a choice of higher intensity levels can solve a problem with considerably fewer number of beams, as compared to a plan with lower intensity levels. The beam numbers in IMRT is also influenced by beam penumbra.^(^ ^10^ ^)^ It is found that for sharper beams with smaller penumbra, the required beam numbers are correspondingly higher. Since beam intensity levels and beam penumbra are taken into account during beamlet intensity optimization by the inverse planning system, the resulting BIPPS curve inherently accounts for their effects. It is to be noted that the algorithms used for beamlet intensity optimization and MLC segmentation can also influence the final beam numbers obtained using the presented method. Though the results obtained in the present study are applicable only to segment‐based IMRT, the method is also applicable to dynamic IMRT.

## V. CONCLUSIONS

The investigation shows that optimization of beam angles certainly reduces the beam numbers required to produce clinically acceptable dose distribution. This can translate into faster and better treatment delivery in terms of number of MUs and segments. The results lead to the conclusion that it is important to understand the interplay between beam angles and numbers for effective use of beam angle optimization algorithms. On the other hand, the algorithms or methodologies employed to find optimal beam numbers should also consider this interplay for choosing beam numbers. We believe that this approach will lead to better and clinically oriented results.

## ACKNOWLEDGMENTS

The authors would like to thank Mr. K. Venkatesan and Mr. K. Ganapathy for their valuable comments on the manuscript.
